# Design of Minimum Nonlinear Distortion Reconfigurable Antennas for Next-Generation Communication Systems

**DOI:** 10.3390/s21072557

**Published:** 2021-04-06

**Authors:** Germán Augusto Ramírez Arroyave, Antoni Barlabé, Lluís Pradell, Javier Leonardo Araque Quijano, Bedri A. Cetiner, Luis Jofre-Roca

**Affiliations:** 1 Department of Signal Theory and Communications (TSC), School of Telecommunications Engineering, Universitat Politècnica de Catalunya (UPC), Campus Nord, 08034 Barcelona, Spain; antoni.barlabe@upc.edu (A.B.); lluis.pradell@upc.edu (L.P.); 2 Department of Electrical and Electronic Engineering (DIEE), Faculty of Engineering, Universidad Nacional de Colombia, Ciudad Universitaria, Bogotá 111321, Colombia; jlaraqueq@unal.edu.co; 3 Electrical and Computer Engineering Department, Utah State University, 4120 Old Main Hill, Logan, UT 84322-4120, USA; bedri.cetiner@usu.edu

**Keywords:** reconfigurable antennas, reconfigurable parasitic layers, antenna optimization, antenna design, nonlinear characterization, behavioral modelling, x-parameters, PIN diode

## Abstract

Nonlinear effects in the radio front-end can degrade communication quality and system performance. In this paper we present a new design technique for reconfigurable antennas that minimizes the nonlinear distortion and maximizes power efficiency through the minimization of the coupling between the internal switching ports and the external feeding ports. As a nonlinear design and validation instance, we present the nonlinear characterization up to 50 GHz of a PIN diode commonly used as a switch for reconfigurable devices in the microwave band. Nonlinear models are extracted through X-parameter measurements supported by accurate calibration and de-embedding procedures. Nonlinear switch models are validated by S-parameter measurements in the low power signal regime and by harmonic measurements in the large-signal regime and are further used to predict the measured nonlinearities of a reconfigurable antenna. These models have the desired particularity of being integrated straightforwardly in the internal multi-port method formulation, which is used and extended to account for the power induced on the switching elements. A new figure of merit for the design of reconfigurable antennas is introduced—the power margin, that is, the power difference between the fed port and the switching elements, which combined with the nonlinear load models directly translates into nonlinearities and power-efficiency-related metrics. Therefore, beyond traditional antenna aspects such as port match, gain, and beam orientation, switch power criteria are included in the design methodology. Guidelines for the design of reconfigurable antennas and parasitic layers of minimum nonlinearity are provided as well as the inherent trade-offs. A particular antenna design suitable for 5G communications in the 3.5 GHz band is presented according to these guidelines, in which the specific switching states for a set of target performance metrics are obtained via a balancing of the available figures of merit with multi-objective separation criteria, which enables good control of the various design trade-offs. Average Error Vector Magnitude (EVM) and power efficiency improvement of 12 and 6 dB, respectively, are obtained with the application of this design approach. In summary, this paper introduces a new framework for the nonlinear modeling and design of reconfigurable antennas and provides a set of general-purpose tools applicable in cases beyond those used as examples and validation in this work. Additionally, the use of these models and guidelines is presented, demonstrating one of the most appealing advantages of the reconfigurable parasitic layer approach, their low nonlinearity.

## 1. Introduction

Wireless communications systems using medium and high transmitter power and advanced modulation techniques must ensure that nonlinearities are below certain safety levels as harmonic components and intermodulation products can degrade system performance and even damage components.

In particular, for New Radio (NR), the Fifth Generation of Mobile Communications (5G), very stringent performance requirements in terms of signal quality, unwanted emissions, and intermodulation are specified [1,2,3,4]. These requirements are accompanied by conducted and radiated testing for verifying conformance to the standard for transmitting and receiving User Equipment (UE)s and Base Station (BS)s [5,6].

As power handling is higher in the BS, attention is focused on this component for which the values specified in [4] are taken as reference. The BS output power limit for conducted tests (defined at antenna connector, or the Transceiver Array Boundary (TAB) connector depending on the BS type) for the cell coverage scenarios considered are (i) Wide Area (No upper limit), (ii) Medium-Range ≤38dBm, (iii) Local Area ≤24dBm.

On the side of signal quality, the metric of interest is the Error-Vector Magnitude (EVM), defined according to the modulation scheme used—QPSK 17.5%, 16QAM 12.5%, 64QAM 8%, 256QAM 3.5%. On the other hand, the limit on unwanted, out-of-band and spurious, emissions is given on terms of two additional metrics, the Adjacent Channel Leakage power Ratio (ACLR) and the Operating Band Unwanted Emissions (OBUE). Of these two, the former is the most stringent with a limit of 45 dB. Finally, on the side of intermodulation, the requirements are that the Inter-Modulation Products (IMP) be attenuated by at least 30 dB.

These metrics and performance requirements imply stringent design goals, but even with a careful engineering some undesired nonlinearities can still persist; therefore, nowadays it is common the use of digital compensation techniques to mitigate the effects of the analog components to achieve conformance to the technical specifications. For these compensation techniques to be effective, a nonlinear model of the analog device to be compensated for is required [7,8].

Even though antennas are traditionally reckoned as inherently linear devices, the recent evolution of the field has relied on introducing nonlinear loads and components such as integrated switches, varactors, and PIN diodes into or in the surroundings of the (re-)radiating structure. This evolution has raised concerns on how these new kinds of nonlinear devices can impact system performance. Consequently, accurate nonlinear antenna modeling is a need that must be addressed, bearing a twofold advantage, as a component design tool and as input for RF system designers to integrate the antenna into the transceiver chain.

The use of PIN diode switches in microwave radio front ends is widespread, and new applications are rapidly emerging, for example in Reconfigurable Antenna (RA) [9,10], reconfigurable Parasitic Layer (PL) [11], reconfigurable surfaces [12,13], reconfigurable phased arrays and a variety of sub-systems for RADAR and Millimeter Waves (mmW) communications. Notwithstanding that PIN diodes are nonlinear components, their system impact on figures of merit such as EVM, and Inter-Modulation Distortion (IMD) is generally overlooked or shadowed by elements conventionally believed to be more critical such as power amplifiers (PA). But when the number of PIN diodes grows or when placed in high power paths, their contribution to system nonlinearities should be accounted for.

Although the initial design and proof of concept of reconfigurable devices can rely on models such as the ideal switch abstraction, linear circuit equivalents, or measured S parameters, the validity and accuracy of these models are rapidly lost as biasing, frequency, and power are changed. Therefore, in stringent applications such as 5G, nonlinear models of the PIN diode should be used to predict system behavior in different operation conditions.

Nonlinear models of microwave devices and circuits [14] can be categorized either as Compact/Physical (based on the physics governing the device) or Black-Box/Behavioral (based on the characteristics of the device from its terminals) [15]. When the internal structure of the Device Under Test (DUT) is not disclosed or is of no interest to the system designer, black-box modeling is the most suitable approach, with various alternatives available [16,17,18,19,20,21].

The X parameters [22] stand out among the available behavioral modeling choices. They are an appealing option given some of their features such as extraction stability, time-invariance, convergence to the S parameters in the small-signal regime, and their integration into simulation software.

Albeit the nonlinear behavior of the PIN diode is widely reported and mostly attributed to its nonlinear resistance characteristic [23,24,25], and SPICE models suitable for the microwave range have been proposed [26,27], most manufacturers do not provide the parameters and values needed to be used in compact models at high frequencies.

Consequently, X parameters are proposed in this work to experimentally obtain a behavioral model of a PIN diode commonly used for mmW applications. The extracted diode model is validated through independent S parameters and harmonic distortion measurements.

Reconfigurable antennas in general and parasitic layer based reconfigurable antennas [11] presenting compact size, low complexity, low power consumption, and small nonlinearities, in particular, are an appealing alternative for the deployment of the 5G-NR usage scenarios (Ultra-Reliable and Low Latency Communications (URLLC), Enhanced Mobile Broadband (EMB), and Massive Machine Type Communications (MMTC)).

**Figure 1:**
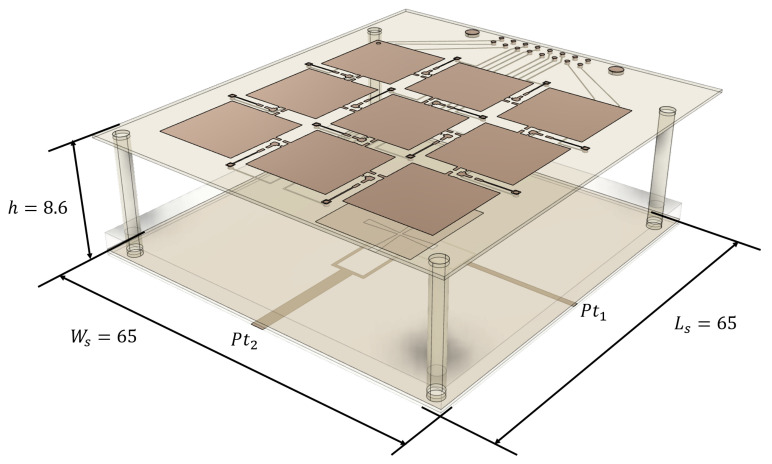
Exploded view of the Parasitic-Layer-based Reconfigurable-antenna used as case study. (Units in mm).

The extracted nonlinear model of the PIN diode switch is a useful input to gain insight into the nonlinear behavior of reconfigurable antennas using PIN diode switches like the one illustrated in Figure 1, based on [28], which is taken as a case of study in this work to demonstrate a new methodology for the design of minimum nonlinearity reconfigurable antennas and parasitic layers suitable for BS and Customer-Premises Equipment (CPE) in 5G EMB scenarios.

The nonlinear behavior of RAs is scarcely reported in the literature, and design strategies considering nonlinearities are non-existent. Two recent experimentally-oriented works are [29], where Third Order Intercept (TOI) and 1 dB Compression Point (1dB CP) are presented, and [30] which focuses on EVM and IMP measurements. Nevertheless, both cases lack an analytical formulation to explain or estimate the nonlinear behavior of the antenna, which would be required to calculate the measured effects or to consider the nonlinearities in the design stage. This is precisely the gap that this work intends to fill, opening new possibilities for the well-balanced design of reconfigurable antennas for the rigorous 5G performance requirements.

Notwithstanding the lack of theoretical approaches and the scarcity of experimental works studying nonlinear effects in RA, the case of nonlinearly loaded basic antennas and arrays has been tackled to some extent. With few exceptions that deal with the nonlinear problem entirely within the electromagnetic solution, which proves useful in cases where nonlinearities are inherent to the antenna or propagation medium materials, the most reasonable approach to the analysis of nonlinearly loaded antennas is to split the problem into an electromagnetic (linear) and a circuital (nonlinear) problem. The former is commonly dealt with by Full-Wave Electromagnetic (FWEM) numerical techniques, whereas the latter can be tackled by formulations such as the Harmonic Balance (HB) analysis [31].

Nonlinear antenna analysis can be traced back to the works of [32], based on a Time-Domain (TD) formulation of Method of Moments (MoM) that allows the direct inclusion of the nonlinear load model into the calculation. Another approach is presented in [33,34], where the antenna problem is solved by MoM, while a Frequency-Domain (FD) formulation based on a Modified Volterra Series (MVS) is used to solve the resulting nonlinear circuit for single and multiple antenna cases. Likewise, Ref. [35] presents a direct nonlinear space-time solution of the whole antenna current leading to limited applicability and an FD solution of the antenna problem followed by a circuital nonlinear time-marching solution. Harmonic balance to solve the nonlinear problem is introduced in [36] alongside a transformation matrix technique in the frequency domain to deal with the nonlinear harmonic load case. In [37], the reflection algorithm [31] and a variation of HB where the direct solution is replaced by Generalized Minimal Residual Method (GMRES) are used. Likewise, in [38], the Arithmetic Operator Method (AOM) is used to deal with the nonlinearity of the load.

A common drawback in these works is that they assume a given simplified model for the nonlinear switching element i−v characteristic which is not often accurate for real-life devices neither directly measurable for RF and microwave components.

Therefore, given the nonlinear characteristics of the loads, a suitable modeling technique must be used. Measured X-parameters of the PIN diode are developed in this work as a convenient alternative for stating the characteristics of the nonlinear loads.

Likewise, the Internal Multi-Ports Method (IMPM) [39,40,41] is further extended in this work to efficiently account for the power delivered to the switching elements and the fed ports. The IMPM requires only one FWEM simulation to determine the behavior of all the possible switches configurations, and for the present application, its use involves placing an antenna port during FWEM simulation on the locations where the switching elements may be placed. This approach employs efficient analytical formulations to perform all the subsequent circuital calculations for the switching states of interest, thus allowing to study reconfigurability attending performance both of the port match and the radiated fields. By combining this technique with the nonlinear models of the diode, estimates of system parameters like the EVM and IMD can be made, allowing the design of minimum nonlinearity reconfigurable antennas and parasitic layers.

The remainder of this paper is organized as follows—Section 2 presents the nonlinear diode characterization and validation as well as the application of the extracted models to a particular case, Section 3 introduces the criteria for the minimum nonlinearity design of reconfigurable parasitic layers alongside novel design guidelines for the implicit trade-offs, Section 4 shows the application of the design guidelines for a particular design of a minimum nonlinearity PL-enhanced antenna; finally, the conclusions section summarizes the main contributions of this work and the possible future developments.

## 2. Nonlinear Switch Characterization

The PIN diode MACOM MA4AGBLP912, operating over the 50 MHz–40 GHz frequency range, was selected for testing. The measurements are performed using a Keysight N5245A PNA-X network analyzer capable of extracting X parameters, connected by means of a Cascade-Microtech 150 µm-pitch Ground-Signal-Ground (GSG) Co-Planar Waveguide (CPW) probe to the test fixture.

### 2.1. Interpretation of X-Parameters

The notation used in X parameters is based on port, harmonic and propagation direction convention, in which $A_{qn}$ represents the nth harmonic of an incoming signal at port *q*, while $B_{pm}$ is the mth harmonic of an outgoing signal at port *p*.

In the general X-parameters formulation, outgoing port waves are expressed as a function of the Large Signal Operating Point Stimuli (LSOPS), which is composed of the device DC Stimulus (DCS), and the RF Stimuli, which in turn is composed of the fundamental magnitude $|A_{11}|$, and the harmonic set of incoming signals $A_{qn}$ which are conveniently referred to the phase of the fundamental $P=e^{j\phi 11}$ ($\phi 11=∠A_{11}$).

A common approximation, valid for most devices, that significantly reduces the computational resources required for measurement, is to consider that the fundamental is the only large RF signal entering the DUT, hence, the only one that can drive it into a nonlinear behavior, while the injected/reflected signals at the remaining ports and harmonics combinations are considered small-signal components. Accordingly, the reference LSOPS is solely defined by $refLSOPS=\{\left\{DCS_{q}\right\},|A_{11}|\}$.

Then, with the aid of linearization and harmonic superposition assumptions, dropping the dependence on refLSPOS for the sake of clarity, the port waves can be related [42] through:

(1) $$B_{pm}=X_{pm}^{\left(FB\right)}P^{m}+\sum_{\left(q,n)\neq (1,1\right)}X_{pm,qn}^{\left(S\right)}A_{qn}P^{m-n}+\sum_{\left(q,n)\neq (1,1\right)}X_{pm,qn}^{\left(T\right)}A_{qn}^{*}P^{m+n},$$

where $X_{pm}^{\left(FB\right)}$, $X_{pm,qn}^{\left(S\right)}$, and $X_{pm,qn}^{\left(T\right)}$ are the X-parameters of type FB, *S*, and *T* respectively, whose interpretation is as follows: the $X_{pm}^{\left(FB\right)}$ represent the large-signal part of $B_{pm}$, and are a set of mappings from $A_{11}$ to the output waves at port *p* and harmonic *m* for a system perfectly matched at the output port and perfectly matched at each harmonic at all ports. The $X_{pm,qn}^{\left(S\right)}$ and $X_{pm,qn}^{\left(T\right)}$ terms determine the sensitivity to mismatch of the system, relating the contributions of the small harmonic-signals from the port *q* at harmonic *n* to the outgoing wave at port *p* and harmonic *m*.

The X-parameters model states that the set of outgoing port waves (for all ports and frequencies combinations) result from a linear mapping of the set of incoming port waves (for all ports and frequencies combinations), but in contrast to S-parameters, not only signal ratios are combined, but additionally this modeling considers the contributions depending on the amplitude of the RF Stimulus (RFS) and the relative phases of the remaining incoming signals (harmonics and intermodulation products) with respect to this reference stimulus.

### 2.2. CPW Test Fixture

**Figure 2:**
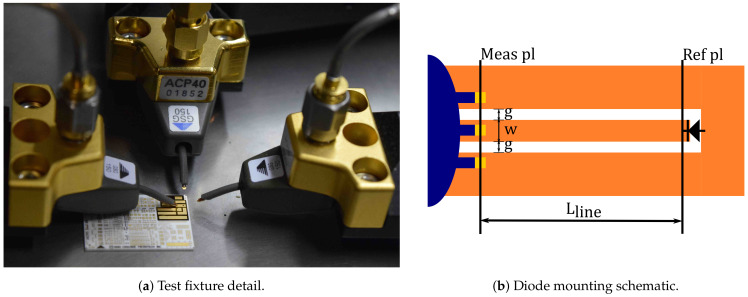
X-parameters measurement setup.

The test-fixture used is supported on an Alumina substrate ($\epsilon_{r}=9.6,\tan\delta =0.0004$, stable up to 67 GHz), and consists of a CPW transmission line with strip width w=0.1mm, gap g=0.05mm, and length $L_{Line}=1.175\;\mathrm{mm}$ from the GSG probe contact plane to the device reference plane, wherein the diode under test is mounted with its cathode connected to the CPW strip and anode connected to ground as is shown in Figure 2. This setup is representative of a PIN diode switch on a reconfigurable antenna when modelled as an internal port where reflection coefficient is of interest.

In order to proceed with the extraction of the diode model, a broadband Short-Open-Load-Thru (SOLT) calibration is first performed, followed by input power calibration and harmonics phase reference calibration as is required for the measurement of X parameters [42]. The diode is biased using an external DC-power supply connected to a built-in biasing-tee of the network analyzer. Subsequently, the diode S parameters are measured, retaining only the reflection coefficient.

As the SOLT calibration reference plane is defined on the GSG probe-CPW line contact, a de-embedding procedure is applied to remove the contribution of the CPW line from the measured diode S parameters and retrieve the actual diode reflection coefficient. A model for the CPW-*even* mode accounting for dispersion, obtained with the aid of accurate 2.5D electromagnetic simulation using Keysight Momentum, is used for that purpose.

Considering the diode mounting and that the X-parameters measurement system used in this work requires the calibration and registering of both ports, some comments on the validity of the measurements are deemed convenient.

Although in the case of S parameters, it is clear that two- and one-port S-parameter measurements would yield the same reflection coefficient, this may not be evident in the case of X-parameters, as in general, the nonlinear functions involved depend on the incoming signals from all the ports/harmonics combinations. However, as in the test fixture used for diode characterization both ports are physically uncoupled, the waves injected at port 2 do not affect the measurement at port 1, hence, inter-port wave dependence vanishes. This is expressed by the reduced one port X-parameters

(2) $$B_{1k}=X_{1m}^{\left(FB\right)}P^{m}+\sum_{n\neq 1}X_{1m,1n}^{\left(S\right)}A_{1n}P^{m-n}+\sum_{n\neq 1}X_{1m,1n}^{\left(T\right)}A_{1n}^{*}P^{m+n},$$

which can be posed in matrix form, allowing the integration of the results into further circuital formulations such as harmonic balance calculations.

(3) $$B_{1}=PX_{1}^{\left(FB\right)}+PX_{1}^{\left(S\right)}{\left(P^{\prime }\right)}^{-1}A_{1}+PX_{1}^{\left(T\right)}P^{\prime }A_{1}^{*},$$

where $B_{1}={\left[B_{11}\cdots B_{1N}\right]}^{T}$, $P=diag(P^{m})$, $P^{\prime }=diag\left(P^{m+1}\right)$, $X_{1}^{\left(FB\right)}={\left[X_{11}^{\left(FB\right)}\cdots X_{1N}^{\left(FB\right)}\right]}^{T}$, $A_{1}={\left[A_{12}\cdots A_{1N}\right]}^{T}$, and $X_{1}^{\left(S\right)},X_{1}^{\left(T\right)}$ are matrices whose elements are of the form $X_{1m,1n}^{\left(\cdot \right)}$.

### 2.3. Measurements

Extensive measurements of S- and X-parameters of diode MA4AGBLP912 were performed in the 1–50 GHz range, using different biasing levels. Note that in the case of the PIN diode, the biasing current $IDC_{1}$ is equivalent to the $DCS_{q}$ term (part of the LSOPS) in the X-parameters notation.

**Figure 3:**
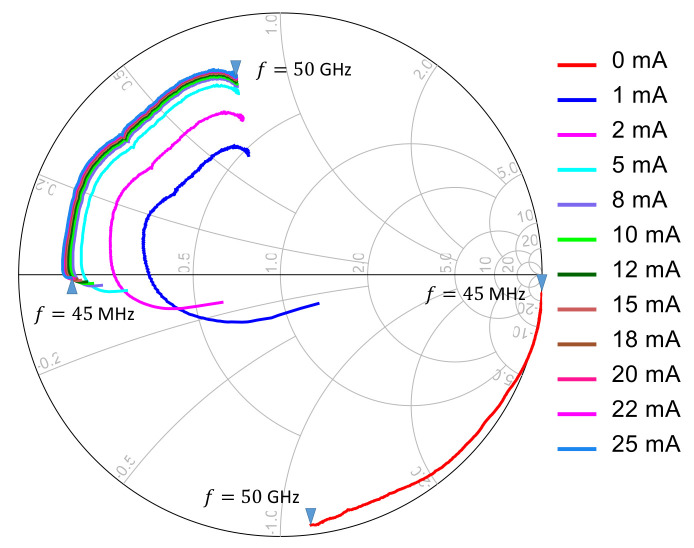
Measured S-parameter (de-embedded) vs. Bias.

Measurements of $S_{11}$ from 45 MHz to 50 GHz are shown in Figure 3. From this figure, it is clear that in the forward bias condition varying DC level has an important impact on the diode behavior. Likewise, it can be observed that for biasing currents higher than 15 mA the diode achieves a stable behavior. Therefore, 0,1 and 20 mA bias are taken as representative of the diode “OFF”, “NL” (nonlinear) and “ON” states. Measurement results are validated against S-parameter files provided by the manufacturer (up to 30 GHz) ascertaining a very good agreement.

One-port X-parameter measurements were carried out at different biasing levels $I_{b}=\left\{0,1,2,5,10,20\right\}$ mA. For each of these DCS a power-frequency grid for $|A_{11}|$ is defined, varying input power in the range $P_{in}=\left\{-10,-6,-2,2,6,10\right\}$ dBm and sweeping fundamental frequency from 1 to 16 GHz with $n_{f}=16$ points, considering up to the third harmonic (total frequency expanding from 1 to 48 GHz). That is, in total there are 6 sets of measurements each with a total of 3 X parameters over a 6×16×3 sampling grid. In contrast, a conventional S-parameter measurement would only contain 6 sets of $n_{f}$ points each.

**Figure 4:**
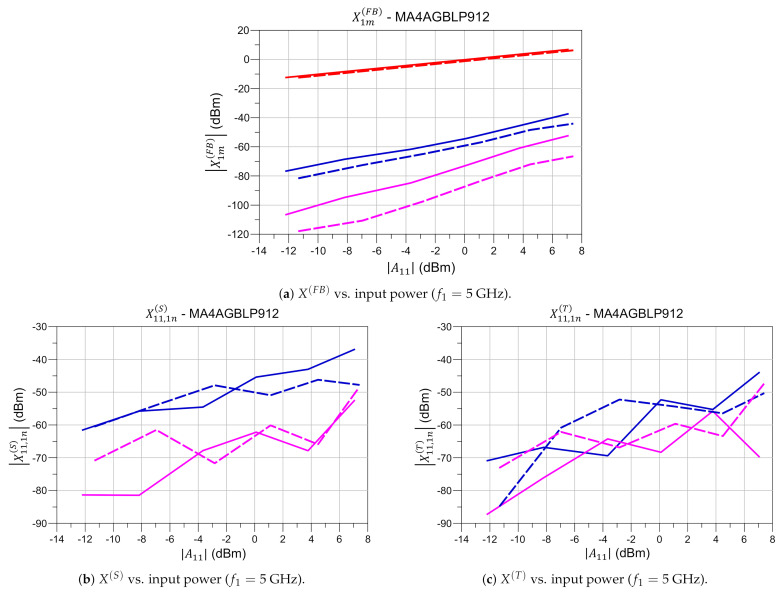
X-Parameters variation with input power at fundamental *f*_1_ = 5 GHz. (⚊ *f*_1_, ⚊ 2*f*_1_, ⚊ 3*f*_1_, ⎯ “Off”, --- “On”).

To ease the comparison of X parameters for the different diode biasing levels, slices of $X_{1m}^{\left(FB\right)},X_{11,1n}^{\left(S\right)}$, and $X_{11,1n}^{\left(T\right)}$ can be taken either at fixed frequencies or input powers of the fundamental $A_{11}$. For example, slices representing the variation with input power at a fundamental frequency $f_{1}=5$ GHz are illustrated in Figure 4 for the diode “ON” and “OFF” states.

Valuable information about the nonlinearity of the DUT can be extracted from the variation with RFS power of the large signal response $X_{1m}^{\left(FB\right)}$, represented in Figure 4a, where it can be observed that for the “OFF” and “ON” states the fundamental and the harmonic tones linearly increase with input power. This figure also demonstrates that the diode is not driven into a high nonlinearity region for the powers used in the model extraction, as in both cases the second and third harmonics are at least 40 dB below the fundamental level. Besides, the $X_{1m}^{\left(FB\right)}$ terms allow an estimation of the Third Harmonic Intercept Point (IP3) that is ∼35dBm in the case of the “OFF” state, and ∼43dBm for the “ON” state.

Another common approach to estimate the nonlinearity of the DUT is by defining the nonlinear transition as the input power resulting in the 1dB CP of the reflected fundamental wave; this value can be extracted directly from Figure 4a or by representing the large signal reflection coefficient $X_{11,11}^{\left(S\right)}\equiv X_{11}^{\left(FB\right)}/\left|A_{11}\right|$. In the diode case, it is apparent that for the input powers used, compression does not occur for the “OFF” nor the “ON” states. In contrast, for the case of the “NL” state, 1 dB CP occurs at around 6 dBm.

On the other hand, the variation with input power of the set of functions $X_{11,1n}^{\left(S\right)}$ illustrated in Figure 4b and $X_{11,1n}^{\left(T\right)}$ illustrated in Figure 4c, indicate a small contribution of the reflected second and third harmonics to the total response at fundamental for both the “OFF” and “ON” diode states.

**Figure 5:**
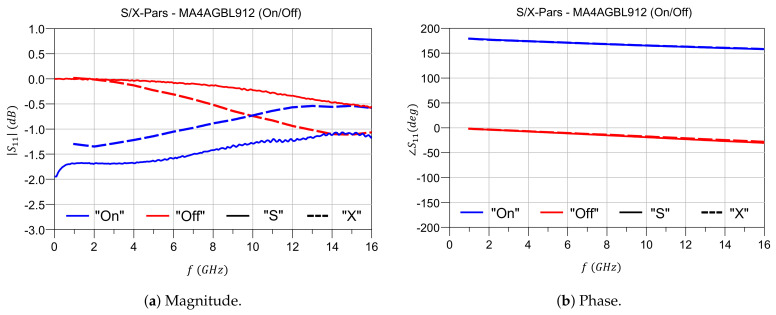
S- and X- parameters small signal convergence.

The X parameters convergence to the S parameters in the small-signal regime is verified for both the “ON” and “OFF” states. Direct S-parameter measurements are compared to X-parameter reduced to S parameters using circuit simulation in Keysight ADS. Results are reported in Figure 5. There is a very good agreement between the two kinds of parameters with maximum magnitude and phase deviation of 0.5dB and $2.5^{\circ }$ respectively.

A further validation was performed contrasting $X_{1m}^{\left(FB\right)}$ with an independent measurement using a spectrum analyzer at ($DCS_{q}=\left\{0,1,20\right\}\;\mathrm{mA}$, $P_{in}=\left\{-2.5,5\right\}\;\mathrm{dBm}$, $f_{1}$ =1–16 GHz). A fundamental to second harmonic rejection above {45,13,55} dB for the three biasing levels was observed at $P_{in}=5\;\mathrm{dBm}$, these values compare well with those observed from $X_{1m}^{\left(FB\right)}$ of {49,13.5,52} dB at $P_{in}=6\;\mathrm{dBm}$ for the same biasing levels.

### 2.4. Nonlinear Assessment of RA and PL

The extracted diode model based on the X-parameters measurements, once validated, becomes a fundamental input to assess the possible nonlinear behavior of reconfigurable antennas and parasitic layers from an analytical viewpoint, and ultimately, a valuable piece of information to be further used as a design tool in the case of minimum nonlinearities reconfigurable antennas and parasitic layers.

**Figure 6:**
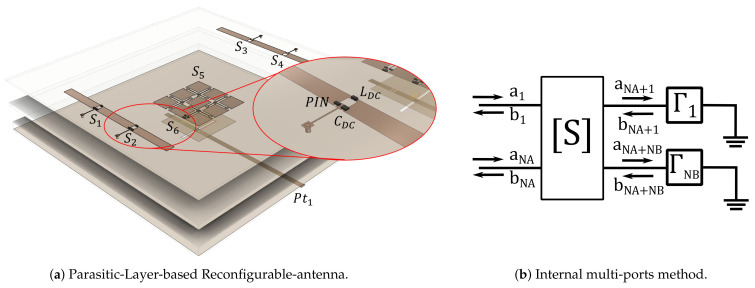
Parasitic-Layer-based Reconfigurable-antenna and antenna equivalent circuit.

To illustrate the use of the X-parameters in a particular case, the antenna reported in [30] and reproduced in simplified manner in Figure 6a is taken as case of study. This antenna uses a total of 17 switches based on the MA4AGFCP910 PIN diode, of which the most critical is the one located in the main feed path ($S_{6}$ in the diagram); likewise, the set of 12 diodes in the intermediate PL ($S_{5}$) are jointly (de)activated, hence, although individually not as significant as $S_{6}$, when combined, their impact on nonlinearity is similar or can be even larger. The switches $S_{1-4}$ providing beam-steering are loosely coupled to the fed power, therefore, their impact on antenna nonlinearity is marginal.

Notwithstanding that this antenna has 6 diverse modes of operation allowing for beam-steering and bandwidth reconfigurability, given the antenna symmetries and the expected small contribution to nonlinearity of the switches on the upper PL, only modes $M_{1},M_{4}$ providing bandwidth reconfigurability are considered for analysis. Switch states for these modes are summarized in Table 1.

As mentioned in the introduction, a common approach for the analysis of nonlinearly loaded antennas is to split the problem into an electromagnetic (linear) and a circuital network (nonlinear) problem. The first one can be dealt by numerical techniques such as MoM, Finite Elements Method (FEM), Finite-Differences Time-Domain (FDTD), whereas the second one can be tackled by nonlinear formulations like the Harmonic Balance (HB).

The internal multi-port method, illustrated in Figure 6b, which proceeds by replacing each switch location by a simulation port that can be later loaded with the proper switch parameters, and that has been extensively used for the analysis and design of RA and PL, is naturally fitted to translate the electromagnetic problem into a circuital one, in which the use of X-Parameters models of the loads, in combination with nonlinear circuital simulation, allow to calculate the nonlinear metrics of interest. After performing the FWEM simulation using the time domain solver in CST Studio, Keysight ADS is used to carry on the corresponding circuital S-parameters and harmonic balance simulations.

**Figure 7:**
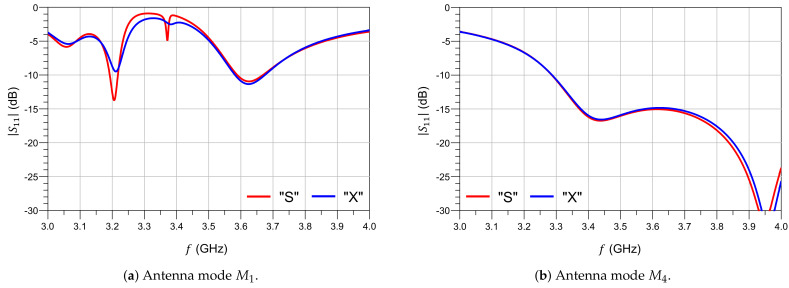
Port match in a test reconfigurable antenna.

Figure 7 shows that in the small-signal regime, the antenna port matching predicted by the X-parameters is quite similar to that predicted by the S-Parameters, ratifying the validity of the approach.

**Figure 8:**
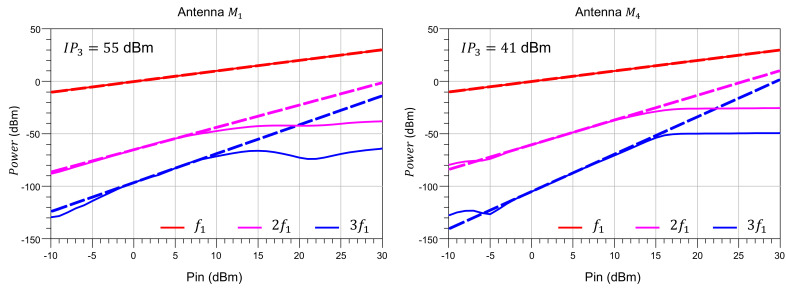
Harmonic components generated by the test reconfigurable antenna, with indication of $IP_{3}$. (**left**) $M_{1}$, (**right**) $M_{4}$.

Afterwards, two relevant metrics to assess the nonlinearity of the antenna are determined by means of an HB simulation in which the input power is swept from −10 to 30 dBm. Figure 8 shows the harmonic components generated by the switches as seen from the antenna input port. From this variation of fundamental and harmonics with input power, the TOI of the antenna can be estimated to be $IP_{3}=$ 55 dBm and 41 dBm for the $M_{1}$ and $M_{4}$ operation modes, respectively.

One step further, the Error Vector Magnitude can also be derived with the aid of the X-parameters nonlinear model. The EVM is a communications performance metric [43] that gained relevance in favor of traditional ones such as Bit-Error Ratio (BER) because of its independence on the receiver (no need to decode the received bit-stream), and its independence on the modulation scheme used [44] in either high- or low-distortion environments.

The EVM is related to transmitter imperfections [45] such as modulator carrier leakage, IQ mismatch, nonlinearity, local oscillator phase noise and frequency error. An approach to calculate the EVM on nonlinear devices is presented in [46], assuming one system impairment is dominant whereas the remaining are modelled as part of the Gaussian noise.

In general, the EVM is defined as

(4) $$\begin{array}{cc} EVM & =\frac{\frac{1}{N_{S}}\sum_{n=1}^{N_{S}}{\left|S_{r}\left(n\right)-S_{t}\left(n\right)\right|}^{2}}{P_{0}}\\ P_{0} & =\frac{1}{N_{S}}\sum_{n=1}^{N_{S}}{\left|S_{t}\left(n\right)\right|}^{2}, \end{array}$$

where $S_{t}\left(n\right)$ and $S_{r}\left(n\right)$ are the nth transmitted and received symbols, respectively.

Although it is valid only in “Data-aided receivers” [47], the Signal-to-Noise Ratio (SNR)-based approximation for EVM is proven to hold well for high SNR setups. It can also be used as an upper bound for the real EVM when the received symbols are estimated.

Using a base-band signal model and reasoning similar to that in [46], the EVM can be expressed in terms of the SNR and the in-band nonlinear distortion (HD). Therefore, acknowledging that the major contribution to the in-band nonlinear distortion comes from the third order intermodulation products [45], the approximated EVM can be expressed as:

(5) $$\begin{array}{cc} EVM & \approx \sqrt{\frac{1}{SNR}+HD}\\ EVM & \approx \sqrt{\frac{N_{0}}{P_{0}}+{\left(\frac{P_{0}}{IP_{3}}\right)}^{2}}, \end{array}$$

which states that in low input power setups, the main driver of EVM is the system noise, while in high power setups the harmonic distortion is the main responsible for error.

**Figure 9:**
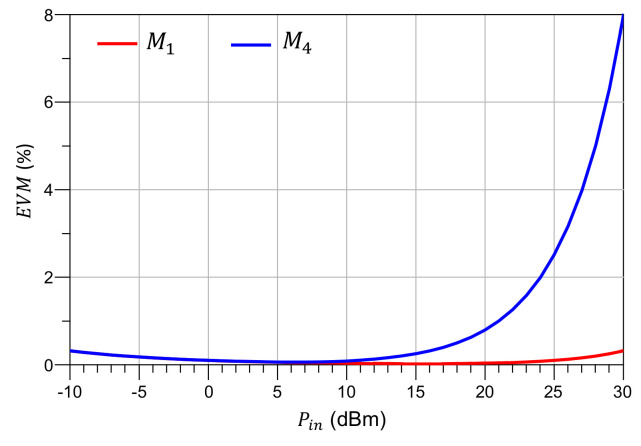
Error Vector Magnitude (EVM) of test reconfigurable antenna.

The approximated EVM is calculated using the results of the HB simulation of the PL-based RA under test, and assuming a moderately high noise floor of N=−60 dBm. Results are shown in Figure 9. The observed results are in agreement with the trends and values observed in the measurements reported in [30], where the antenna operation mode $M_{4}$ presents a higher EVM than the $M_{1}$, and the EVM is below 8% for input powers up to $P_{in}=30$ dBm, for all the antenna states of interest. This results also demonstrate that the pl-approach is a low-nonlinearity alternative to achieve complex antenna functions in accordance with the performance limits established for 5G.

## 3. Minimum Nonlinearity PL Design Guidelines

The advanced design of parasitic layers, and reconfigurable antennas in general, heavily relies on optimization techniques to find an adequate set of switching states to achieve a target set of figures of merit. These figures of merit are usually specified in terms of fundamental antenna parameters for example, port parameters, such as match and coupling, and pattern features such as direction of maximum, gain and polarization.

Although nothing prevents the inclusion of system-related performance metrics impacted by the antenna, such as coverage, throughput, BER or EVM, or any other parameter of interest in particular applications, the use of basic antenna properties is often the most efficient option, as the computational cost of calculating these derived quantities make them impractical when evaluating beyond a few thousands of switch states. This complexity is exacerbated if structural variations of the basic radiator are to be considered in a co-optimization process as for example in [48].

Therefore, the use of basic antenna parameters and system-level performance estimators directly derived through simple calculations may be used whenever possible to predict the better performing configuration when comparing among several different alternatives.

As the main interest of this work is the design of reconfigurable antennas and parasitic layers with minimum nonlinearities and impact on related system-level metrics such as EVM, a partitioning of the design process into three stages is proposed aiming to reduce the computation time and achieving controllable results. The stages of the design methodology are:

- Choose an optimal trade-off starting point for the antenna geometry.
- Determine the optimum switch configurations using the performance metrics vector defined in terms of fundamental antenna parameters derived from small-signal formulations.
- Based on nonlinear load models, accurately calculate the nonlinearities and system-related parameters for the optimum configurations.

**Figure 10:**
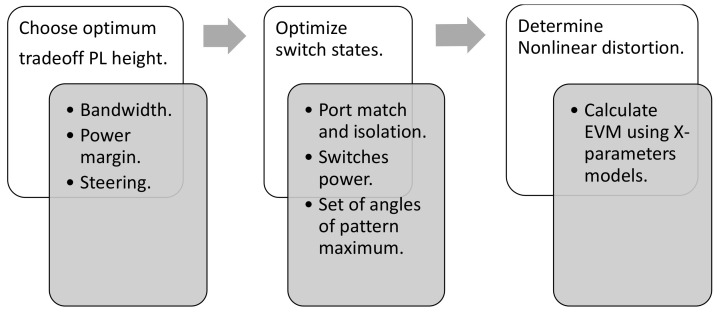
Flow chart with the main elements of the design methodology.

A high level flowchart diagram illustrating the main elements of the proposed design methodology is presented in Figure 10.

### 3.1. Adding New Design Criteria

A general optimization problem is defined as a search in an *n*-dimensional decision space, mapped through a cost function *f* to a lower *k*-dimensionality objective space, in which a comparison of elements *x* in the original space is performed through metrics *y* defined in the destination space. In the case of RA design, diverse antenna parameters compose the comparison metrics, hence, a multiobjective optimization problem is implicit.

(6) $$\begin{array}{c} f:R^{n}\to R^{k}\\ y=f(x). \end{array}$$

Common RA design approaches mix multiple parameters to obtain a single performance indicator to facilitate decision, however, if the cost function is defined in a way such that k=1, there is a risk of over-simplifying the problem as there exists the possibility that *f* be a surjective projection of $R^{n}\to R$ having several individuals with very similar cost values and quite different configuration and performance in the individual criteria; this prevents the discovery of the trade-offs involved in the design process, and may result in the optimization being biased towards the easier of the individual performance metrics, neglecting the harder ones.

Adding dimensions to the objective space allows the creation of Pareto fronts, representing the best objective combination, enabling the visualization of trade-offs and exploring the interrelation between objectives. Furthermore, adding new decision axes to the objective space creates new separation criteria for configurations with very similar performance on the other axes.

In this work, a new power-related metric is defined in addition to the port parameters and far-field related metrics allowing to account for antenna port match, isolation, beam pointing, gain, power efficiency and nonlinearity.

### 3.2. Extension of the IMPM to Account for Nonlinearities

The Internal Multi-Ports Method (IMPM) is an efficient way to characterize the behavior of a reconfigurable device for a set of switch states, requiring only one FWEM simulation at setup. After setup, formulations yielding results equivalent to circuital simulations allow to determine the effects of the terminating loads on the fundamental antenna parameters. A simple extension to the IMPM is added to account for the power delivered to the fed ports and to the switches, becoming a useful indicator of power efficiency and possible excitation of nonlinearities. Expressions to calculate loaded port parameters, radiated field and port power are briefly summarized.

The setup FWEM simulation is performed to obtain the reference network scattering parameters $S\in C^{N_{p}\times N_{p}}$, and the field radiated when the nth port is fed with unit amplitude (and the others are terminated in matched loads) ${\bar{E}}_{n}\left(\theta ,\phi \right),\;n\in \left\{1\cdots N_{P}\right\}$, where $N_{p}=N_{A}+N_{B}$, $N_{A}$, and $N_{B}$ are the number of total, fed (external), and loaded (internal) ports, respectively. The matrices and vectors involved in the formulation can be partitioned to index the external or internal ports, A,B subscripts are used to denote this partitioning.

From the defining relation of the total S-matrix b=Sa in terms of the power wave amplitudes $a_{m}=v_{m}^{+}/\sqrt{2Z_{cm}}$, $b_{m}=v_{m}^{-}/\sqrt{2Z_{cm}}$ ($v_{m}^{\pm }$ is the peak amplitude of the incoming/outgoing voltage wave at the mth port) for ports with possibly different characteristic impedances, the block partition described above results in:

(7) $$\left[\begin{array}{c} b_{A}\\ b_{B} \end{array}\right]=\left[\begin{array}{cc} S_{\mathrm{AA}} & S_{\mathrm{AB}}\\ S_{\mathrm{BA}} & S_{\mathrm{BB}} \end{array}\right]\left[\begin{array}{c} a_{A}\\ a_{B}. \end{array}\right]$$

Noting that only input ports have a source signal $a_{A}$, while for the loaded ports the incoming signals are actually reflections on the load terminations, obtainable from $\Gamma_{B}$, the diagonal matrix telling the reflection coefficient of the terminating loads, we can write:

(8) $$\left[\begin{array}{c} b_{A}\\ b_{B} \end{array}\right]=\left[\begin{array}{cc} S_{\mathrm{AA}} & S_{\mathrm{AB}}\\ S_{\mathrm{BA}} & S_{\mathrm{BB}} \end{array}\right]\left[\begin{array}{c} a_{A}\\ \Gamma_{B}b_{B} \end{array}\right]$$

From the expressions above, we can compute all the unknown wave amplitudes from the known input amplitudes $a_{A}$:

(9) $$\begin{array}{ccc} a_{B} & = & \Gamma_{B}b_{B} \end{array}$$

(10) $$\begin{array}{ccc} b_{A} & = & \left\{S_{\mathrm{AA}}+S_{\mathrm{AB}}{\left(\Gamma_{B}^{-1}-S_{\mathrm{BB}}\right)}^{-1}S_{\mathrm{BA}}\right\}a_{A} \end{array}$$

(11) $$\begin{array}{ccc} b_{B} & = & {\left(I-S_{\mathrm{BB}}\;\Gamma_{B}\right)}^{-1}S_{\mathrm{BA}}\;a_{A}. \end{array}$$

Now, the aggregated radiated field for a particular excitation and load condition can be calculated as follows from both $a_{A}$ and $a_{B}$:

(12) $${\bar{E}}_{tot}\left(\theta ,\phi \right)=\sum_{n\in pts}a_{n}{\bar{E}}_{n}\left(\theta ,\phi \right).$$

A number of quantities of interest can now be obtained from these wave amplitudes:

(13) $$\begin{array}{ccc} P_{in} & = & {∥a_{A}∥}^{2}-{∥b_{A}∥}^{2} \end{array}$$

(14) $$\begin{array}{ccc} P_{sw} & = & {∥b_{B}∥}^{2}-{∥a_{B}∥}^{2} \end{array}$$

(15) $$\begin{array}{ccc} P_{ant} & = & {∥a_{A}∥}^{2}+{∥a_{B}∥}^{2}-{∥b_{A}∥}^{2}-{∥b_{B}∥}^{2} \end{array}$$

(16) $$\begin{array}{ccc} \eta_{tot} & = & \frac{\eta_{ant}P_{ant}}{P_{in}}, \end{array}$$

where $P_{in}$ is the net input power to the reconfigurable antenna system, $P_{sw}$ is the power dissipated by the switches, $P_{ant}$ is the net input power to the elementary radiator (the passive/linear portion of the system), and $\eta_{tot},\;\;\eta_{ant}$ are the efficiencies for the full reconfigurable antenna system and the passive antenna element, respectively.

Under a pure tone excitation, and considering that the antenna building materials and surrounding environment have a linear behavior for all operating circumstances, the only source of nonlinear distortion are the internal port loads and their frequency conversion characteristics.

Although the presented formulation relates only one input frequency to the same output frequency, it can be extended to account for harmonics induced by the nonlinear loads by properly adapting the vectors and matrices involved in the calculations. In particular, if $N_{H}$ is the number of harmonics to consider, the vector quantities in the presented formulation can be expressed as composed by sub-vectors of $N_{H}$ terms each, forming a longer extended vector of size $N_{p}N_{H}$. This implies also the extension of the involved matrices to include the relationships at the harmonic frequencies, therefore each matrix entry will be a sub-matrix of size $N_{H}\times N_{H}$, totaling a size of $\left(N_{p}N_{H}\right)\times \left(N_{p}N_{H}\right)$ for a full $N_{p}$-ports matrix.

In this case, S, which involves the linear antenna portion, will be a matrix composed by diagonal matrices, that is, for any pair of ports (p,q), and harmonics (m,n), $S_{pq}\left(m,n\right)=0$, ∀m≠n.

On the other hand, given the non-linearity of the terminating loads (switches), $\Gamma_{B}$ will be a block diagonal matrix with each block representing a given switch, and all blocks being fully populated; therefore the reflection coefficient of a single load $\Gamma^{l}$ must provide a representation for harmonic frequencies conversion, taking the form

(17) $$\Gamma^{l}=\left[\begin{array}{ccc} \Gamma^{l}\left(1,1\right) & \cdots & \Gamma^{l}\left(1,N_{H}\right)\\ \vdots & \ddots & \vdots \\ \Gamma^{l}\left(N_{H},1\right) & \cdots & \Gamma^{l}\left(N_{H},N_{H}\right). \end{array}\right]$$

From this expression, the convenience of the X-parameter formulation for the loads modelling results evident as it embeds the required frequency conversion as a function of the input power.

As an additional remark, it must be noted that when input power is low enough, the cross-frequency components are negligible and consequently the $\Gamma^{l}$ reduce to diagonal matrices, thus the harmonics calculated are only affected by signals in their same frequency, and the harmonic-extended system is reduced to a linear relationship.

As mentioned earlier, for the optimization stage the interest is on efficiently comparing several thousands of configurations; therefore, a valid indicator of the nonlinear performance of a given switch configuration is adopted instead of solving the nonlinear extended system. For this purpose, the power margin (ΔP) is defined as the difference between the power delivered to the fed port and the power delivered to the switches, over the frequency range where the excited port is well matched:

(18) $$\begin{array}{cc} f_{r} & :\left|S_{ii}\left(f_{r}\right)\right|<-10\mathrm{dB}\\ \Delta P & =\langle \frac{P_{in}\left(f_{r}\right)}{P_{sw}\left(f_{r}\right)}.\rangle \end{array}$$

The definition and use of this metric is supported on the fact that for the “ON” and “OFF” switch states, up to moderate input powers, the power of the fundamental tone is much higher than that of the harmonics, as was confirmed by the X-parameters measurements performed. Therefore, the nonlinearities can be predicted by the switch power at the fundamental frequency. Likewise, as was evidenced for the antenna presented as case of study, although the maximum power through a switch is a significant driver of nonlinearities, the total power delivered to the switches set may be a more determinant factor.

### 3.3. PL Design Trade-Offs

From the expressions obtained above, it can be observed that the nonlinear perturbation comes from the waves reflected by the loads, given by the term $S_{\mathrm{AB}}{\left(\Gamma_{B}^{-1}-S_{\mathrm{BB}}\right)}^{-1}S_{\mathrm{BA}}\;a_{A}$. Accordingly, if the nonlinear effects are to be minimized there are two approaches:

(1) Reducing the $S_{\mathrm{AB}},\;S_{\mathrm{BA}}$ terms, that is, the coupling between external and internal ports. This is the underlying philosophy of the PL as there is a physical separation between the feed ports and the switches. Note that this coupling can be controlled by the PL height above the basic antenna, adding one degree of freedom to the design. However, changing the PL height over the basic antenna also impacts the radiated field and port match, requiring a delicate balance.
(2) Reducing the ${\left(\Gamma_{B}^{-1}-S_{\mathrm{BB}}\right)}^{-1}$ harmonic terms. This could be attained by a careful antenna design that provides an adequate matching between the terms in the subtraction, however, given that normally the minuend will be dominant, this is mostly technology dependent, reinforcing the need for accurately characterizing the switch nonlinearities and the benefits of using these models as a detailed design tool.

To examine the effects of the PL height ‘*h*’ over one basic antenna and its effects on the reconfigurability of parameters like port matching, radiated field, and nonlinearities, the parasitic-layer-based reconfigurable-antenna illustrated in Figure 1 is taken as a reference.

A parametric variation of ‘*h*’ is performed on this reference antenna, aiming to determine a feasible trade-off region which can be used as design guideline, even for other PL pixellations and number of switches, as well as other antenna configurations. Therefore, the interest is in average or expected behavior rather than in particular switch states behavior. Aggregated port match bandwidth, maximum steering angle, and average power margin are used as metrics of comparison.

**Figure 11:**
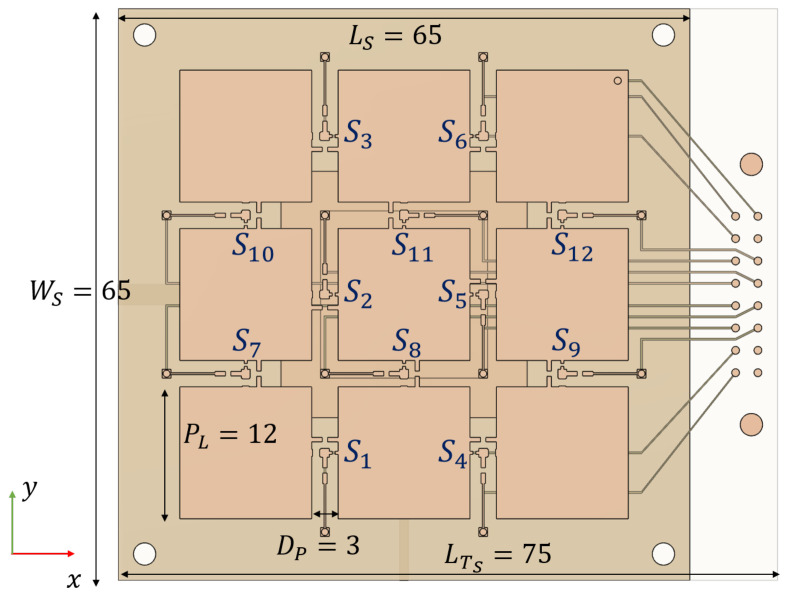
Parasitic-Layer-based Reconfigurable-antenna.

The PL based RA is composed of a basic 2-ports aperture-coupled microstrip patch radiator matched at $f_{0}=3.5$ GHz, on a th=3 mm thick Rogers RO4003 substrate $\epsilon_{r}=3.55$. The PL is a 3×3 pixellation supported on a th=0.51 mm thick Rogers RO4003 substrate, placed at a distance *h* from the basic antenna, with size and switches numbering as illustrated in Figure 11. This design is based on the single-port presented in [28], which was fabricated and measured with good agreement between simulations and measurements, working from 2.4 to 2.5 GHz, providing steering in nine directions $\theta_{0}\in \left\{-30^{\circ },0^{\circ },30^{\circ }\right\},\;\phi_{0}\in \left\{0^{\circ },45^{\circ },90^{\circ },135^{\circ }\right\}$ with an average gain of 6.5 dB.

**Figure 12:**
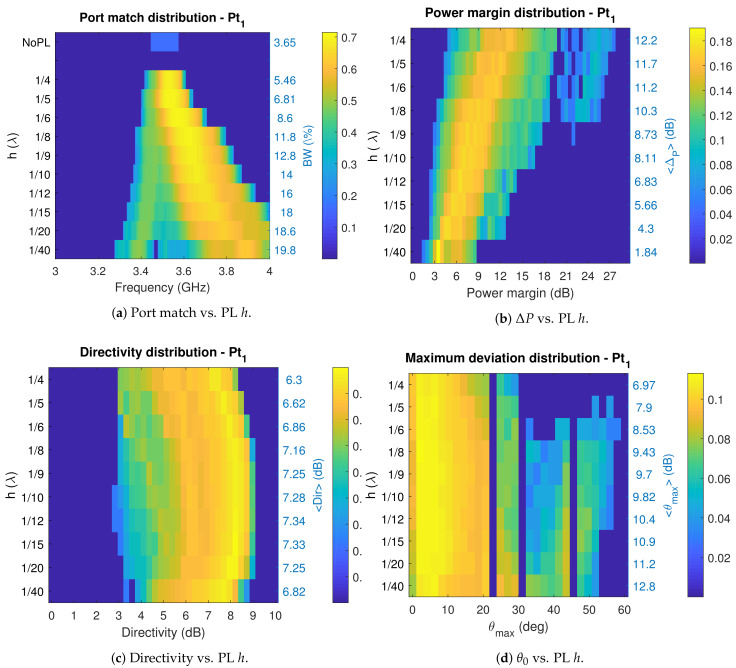
Parasitic-Layer-based Reconfigurable-antenna tradeoffs.

The parametric sweep is performed using the frequency-domain solver of CST studio, for $h=\lambda_{0}/\left\{40,20,15,12,10,9,8,6,5,4\right\}$, retaining for each case the ‘unloaded’ S-parameters file and the radiated fields generated by each ‘port’. In a later step, the IMPM is applied to determine the metrics of interest. As the number of switch configurations is relatively low (4096), exhaustive search is feasible in a short time for each height value. The sweep results for antenna port 1 are summarized in the set of plots of Figure 12 which represent the probability distribution of configurations for a given figure of merit in terms of the variations of the parameter *h*. Results for port 2 are almost identical and are omitted.

The first that can be observed in Figure 12a is that, as expected, the PL loading alters the port impedance in a way that when the separation is smaller, the obtainable bandwidth is higher, and when separation grows, convergence to the bandwidth of the simple antenna is achieved. This is in a good agreement with the experimental results in [40], for a higher complexity PL where a statistical sweep is performed for practical reasons. There can also be appreciated that most configurations are matched at a frequency above that of the basic antenna, suggesting that when starting a new design, the base antenna should resonate at a lower frequency than that of the intended central frequency. An important remark from this figure, is that a reconfigurable bandwidth greater than 10% can be achieved for separations lower than λ/8.

On the other hand, Figure 12b, illustrates the power margin variation with *h*. Although high power margins ∼9 dB can be achieved for some configurations with very narrow separations, average margins in excess of 6 dB are achieved for separations beyond λ/12. However, there must be noted that the average $\Delta_{P}$ increment is not linear, and beyond λ/8 growth is slower.

Variation of directivity with *h* is shown in Figure 12c. Directivity is preferred over gain to isolate the effect of the PL on the shape of the radiated field, without including the effects of port matching. This figure shows that directivity can be improved up to a pair of dBs with respect to that of the basic antenna. Most importantly, for a wide range of height variations (λ/20−λ/6), on average, the effect of the PL does not lead to a degradation of directivity when compared to that of the base antenna.

Finally, the angular variation of the pattern maximum with the PL height over the basic antenna, allows to determine the feasibility of finding patterns with maximum radiation pointing towards specific directions $\left(\theta_{0},\phi_{0}\right)$. A 2D-histogram of the $\left(\theta_{0},\phi_{0}\right)$ distribution was created for each value of *h* to get an exact description of the angular distribution of pattern maximum. From this depiction a trend was observed, and is that the maximum deviation tends to cluster at $\left(\theta_{0},\phi_{0}\right)=\left(45^{\circ },\left\{60^{\circ },120^{\circ },240^{\circ },300^{\circ }\right\}\right),\;\mathrm{and}\;\left(30^{\circ },\left\{0^{\circ },180^{\circ },360^{\circ }\right\}\right)$. Taking this into consideration, a simpler diagram of the maximum θ deviation, for all the possible ϕ values, is created as illustrated in Figure 12d, where configurations with D<6 dB have been filtered out. From this figure, it is apparent that the maximum deviation changes little with height, and that up to λ/5 deviations around $50^{\circ }$ are achievable. However, the angular distribution shows that there are more configurations with larger steering for PL separations in the range λ/20−λ/10.

**Figure 13:**
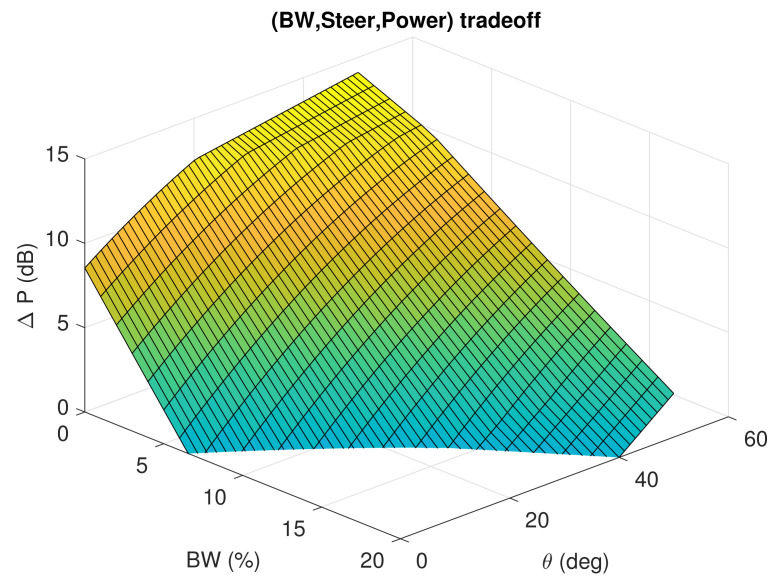
Design Trade-Offs of a Parasitic Layer (PL) based Reconfigurable Antenna (RA).

Finally, aiming to capture the overall reconfigurability potential of the PL-based-RA, and as a general purpose design guideline, the average metrics encountered are expressed by means of a tradeoffs surface as illustrated in Figure 13, showing how much bandwidth reconfigurability, power margin and steering can be expected for a particular setup.

The set of plots of Figure 12 and Figure 13 have presented a general description of the effects of the PL on the fundamental parameters of a basic antenna, and important guidelines for the design as the optimum height region for placing the PL to achieve a desired compromise of objectives.

## 4. Parasitic Layer Design Example

It is foreseen that a large portion of the initial deployments of the 5G-NR will be concentrated on the Freuency Range 1 (FR1) (sub-6 GHz portion of the spectrum) [49], and special interest is placed in the 3.5 GHz band where moderately large bandwidths up to 100 MHz can be allocated. A particular reconfigurable antenna example for the 3.5 GHz band is presented in this section to illustrate the minimum nonlinearities design method developed in the previous section.

The most computationally expensive part of the design process is the initial FWEM simulation to get the ‘unloaded’ antenna parameters, this simulation must be performed for any candidate value of *h* as there is not a straightforward way to reuse simulation results for other *h* values. Accordingly, the presented guidelines for determining the optimum *h* are intended to greatly reduce the design time.

After the fundamental design trade-offs of the reconfigurable parasitic layer are established, an optimum region for the separation between the basic antenna and the PL can be determined based on the compromise that must be made between bandwidth, linearity, and beam-steering. For instance, for 5G applications covering the n78 band at 3.5 GHz, a good starting point should be h=λ/9−λ/12 that can achieve around 12–15% bandwidth, an average 7.3 dB directivity, and a rich beam-steering capability with maximum deviations up to $55^{\circ }$, joined with average power margins in the range 7–9 dB.

However, beyond appropriate region for PL placement, particular indications on how to determine the switch states to achieve the optimum compromise of the objectives are not yet given. The optimum switch states determination is demonstrated by means of a particular design example.

In this case, the same topology used to determine the basic trade-offs is employed with some variations, first the basic antenna is modified to resonate at a lower frequency aiming to have a lower cutoff frequency and a richer number of well-matched configurations at the central frequency of 3.5 GHz, also, the pixel size is increased to 14 mm aiming to have finer control of steering.

The switch state determination is posed as a multidimensional optimization problem, in which a 3D cost function considering S-parameters, beam pointing and switches power, is defined and explored using Pareto fronts to obtain antenna realizations with an optimum objective compromise. The cost function vector is defined as:

(19) $$\begin{array}{cc} J(k) & =\{J_{S}\left(k\right),J_{\Delta P}\left(k\right),J_{\Delta (\theta ,\phi )}\left(k\right)\}\\ J_{S}\left(k\right) & =\max_{f}\left(\max_{i,j}\left(|S_{ij}\left(k,f\right)|\right)\right)\\ J_{\Delta P}\left(k\right) & =-\Delta P(k)\\ J_{\Delta (\theta ,\phi )}\left(k\right) & =\Delta \left(\theta ,\phi \right)\left(k,f_{c},\theta_{0i},\phi_{0i}\right), \end{array}$$

where each component is normalized and mapped to the range [0,1] by the limits $target\;S_{ij}\in \left\{-10,0\right\}$ dB, targetΔP∈{−10,−3} dB, $target\;\Delta \left(\theta ,\phi \right)\in \left\{0^{\circ },10^{\circ }\right\}$. Imposing requirements on port match and coupling, power margin, and angular deviation from target. The set of desired angles is $\left(\theta_{0},\phi_{0}\right)=\left\{\left(0^{\circ },0^{\circ }\right),\;\mathrm{and}\;\{\left(40^{\circ },\left\{0^{\circ },45^{\circ },90^{\circ },135^{\circ },180^{\circ },225^{\circ },270^{\circ },315^{\circ }\right\}\right)\right\}$, therefore $J_{\Delta (\theta ,\phi )}\left(k\right)\in R^{9}$. Further restrictions on results are imposed discarding those configurations with Dir<6 dB.

**Figure 14:**
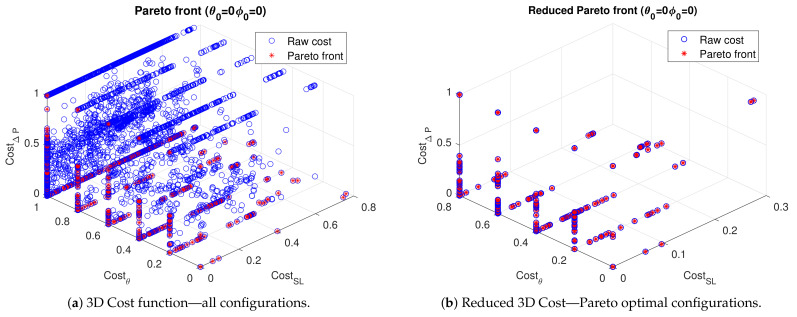
Optimization process.

After each individual is assigned a cost vector, weak Pareto optimality criterion is used to retain only the better performing configurations for each of the desired modes of operation. The process is illustrated for the $\left(\theta ,\phi \right)=\left(0^{\circ },0^{\circ }\right)$ target in Figure 14. The cost of all the possible configurations is shown in Figure 14a, while the reduced set of non-dominated individuals is illustrated in Figure 14b, this set represents the configurations with better performance in any of the objectives defined.

Depending on the reachable cost vector for a particular target mode of operation, and whether a small mismatch, miss-pointing, or higher power going to the switches is tolerable, the designer can evaluate a subset of the configurations on the non-dominated set and determine, based on features like for instance pattern shape, impedance bandwidth, or nonlinear metrics, the configurations that better suits the application.

The procedure illustrated in Figure 14, is carried out for each of the 9 beam directions intended, and for both ports. Although this process can be seen as performing 18 independent optimizations, it is very fast as cost vector is calculated only once for all the switches configurations and the remaining problem is dealt as a classification task repeated for the intended 18 configurations. Results are shown and discussed only for port 1 noting that the values obtained for port 2 are very similar.

Once the better performing configurations are found, these are validated by simulation with CST Design Studio, finding an almost exact match. Similarly, the port parameters calculated using the measured S- and X-parameters models of the loads are verified to be in a very good agreement.

The final configuration of the antenna have a satisfactory performance characterized by an aggregated bandwidth of 10.5% centered at the intended $f_{c}=3.5$ GHz. Additionally, the optimum configurations providing beam-steering, denoted as $M_{1-9}$, are well matched and isolated at the central frequency, albeit slight shifting of minimum is allowed as the balancing criteria privileges accurate pointing over port impedance. The port match and isolation at $f_{c}$ are summarized in Table 2.

On the other hand, the power margin for the optimum configurations are (13.37, 5.50, 7.66, 7.75, 6.98, 5.52, 7.04, 7.66, 6.94) dB, demonstrating a mean value of 8.34 dB, what is a 6 dB improvement when compared with a design strategy that only considers port match and radiation pattern characteristics, and is in accordance to the expected value from the design guidelines, this means also that a small fraction of the power entering the antenna is dissipated on switches and thus a low nonlinearity is expected.

**Figure 15:**
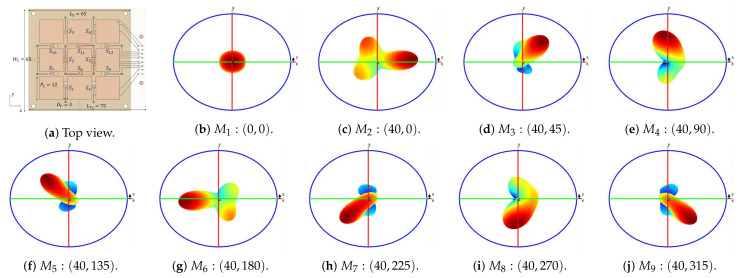
Radiation diagrams at Port 1, for operating mode *M_i_*, (*i* ∈ 1 ... 9), pointing towards (*θ*_0_, *ϕ*_0_).

Likewise, Figure 15 shows the radiation diagrams for the final antenna configurations for port 1. From this figure, a very good pointing towards the target angles defined is observed. Also, a mean gain of 6.13 dB is found which is in accordance with previous PL designs.

**Figure 16:**
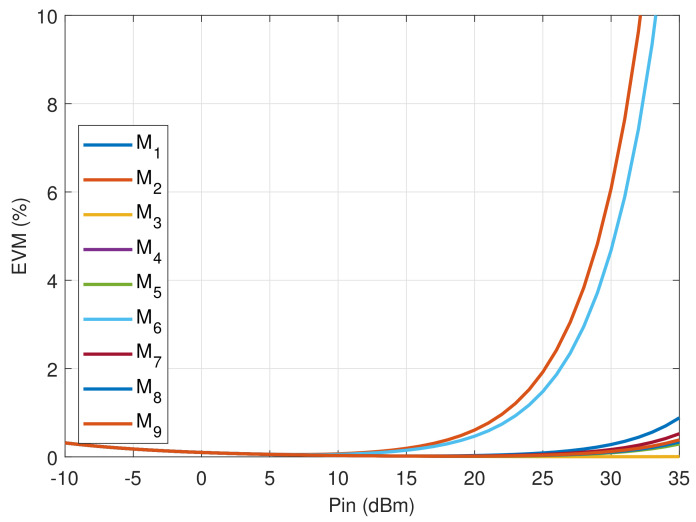
EVM of the Minimum Nonlinearity Parasitic-Layer-based Reconfigurable Antenna.

Finally, the IP3 and further EVM nonlinear metrics of the antenna optimum configurations are calculated using the diode nonlinear diode models extracted by X-parameters measurements. Results for EVM are illustrated in Figure 16 showing that up to 30 dBm the EVM is below 6% for all the antenna states, and that up to 35 dBm the mean evm is below 4%. In comparison with a design strategy that only considers port match and radiation pattern characteristics, where evm values up to 20% are observed, this is a 12 dB improvement. Note that the modes with the lower power margin present the higher evm as expected from the design assumptions.

This result confirms the suitability of this parasitic-layer-based reconfigurable-antenna design for 5G NR mobile broadband systems, and at the same time shows the benefits of the new design approach considering the switches power to minimize the nonlinear distortion.

Even though, to the best of the authors’ knowledge, this is the first effort to minimize the nonlinear distortion of a reconfigurable antenna, and the design technique has implications beyond the particular results presented as example, a comparison with similar works reported in the literature, in terms of basic antenna parameters, size, and nonlinearities when available, is presented in Table 3.

## 5. Conclusions

A method for the nonlinear characterization of a mmW PIN diode has been proposed. It is based on X-parameters measurements obtained using a test set composed of a nonlinear network analyzer, a one-port on-wafer probe station and a CPW test fixture.

The proposed method has been applied to characterize a commercial PIN diode commonly used in reconfigurable antenna applications spanning a frequency range from 1 to 48 GHz.

The measured X parameters have been validated by good agreement obtained between the harmonic rejection derived from X parameters to independent harmonic measurements, as well as from measurements in the small signal regime, where nonlinear X-parameters converge to measured S parameters.

The X parameters model is used to assess the possible nonlinearities on a reconfigurable antenna, showing consistency with experimental results.

Guidelines for the design of Reconfigurable Antennas in general and particular application to Parasitic Layers have been presented accounting for a new performance metric related to the power efficiency and possible emergence of nonlinearities.

Optimum tradeoffs for the separation of the basic antenna and a reconfigurable parasitic layer to attaining particular objectives related to port match, nonlinearity, and beam-steering have been discussed.

A complete methodology for the design of minimum nonlinearity reconfigurable antennas has been presented, accounting also for port match and beam-steering.

The methodology is based on (i) Determining the optimal PL distance to the basic antenna. (ii) Determine the optimum switch configurations. (iii) Based on nonlinear load models, accurately calculate the nonlinearities and system-related parameters only for the optimum configurations.

A design example of a parasitic-layer-based reconfigurable-antenna suitable for 5G NR communications in the 3.5 GHz band, from the port match, beam-steering and nonlinear distortion is presented.

The particular antenna design is a suitable alternative for 5G-NR EMB usage scenarios in the 3.5 GHz band, bearing 2 ports with a 11% bandwidth, 9 beams pointing in $\left(\theta_{0},\phi_{0}\right)=\left\{\left(0^{\circ },0^{\circ }\right),\;\mathrm{and}\;\{\left(40^{\circ },\left\{0^{\circ },45^{\circ },90^{\circ },135^{\circ },180^{\circ },225^{\circ },270^{\circ },315^{\circ }\right\}\right)\right\}$, and an average realized gain of 6.1 dB. The whole structure fits within a compact size of 0.76×0.87×0.1 wavelengths. The presented antenna has an average Error Vector Magnitude (EVM) of 4% at an input power of 35 dBm.

With the application of this new design methodology an improvement of 12 and 6 dB of the EVM and power efficiency, respectively, were obtained when compared with conventional reconfigurable antenna design strategies.

## Figures and Tables

**Table 1:** Switch states for the antenna modes of interest.

Mode	$S_{1}$	$S_{2}$	$S_{3}$	$S_{4}$	$S_{5}$	$S_{6}$
$M_{1}$	0	0	0	0	0	1
$M_{4}$	0	0	0	0	1	0

**Table 2:** Antenna port parameters at $f_{c}$.

Param	$M_{1}$	$M_{2}$	$M_{3}$	$M_{4}$	$M_{5}$	$M_{6}$	$M_{7}$	$M_{8}$	$M_{9}$
$S_{11}$	−11.95	−7.80	−16.81	−13.19	−12.18	−8.59	−11.95	−12.24	−11.67
$S_{21}$	−14.63	−19.49	−13.32	−10.41	−10.47	−19.39	−11.62	−10.73	−10.25
$S_{22}$	−11.30	−11.53	−13.32	−9.82	−10.24	−12.29	−9.95	−10.39	−11.37

**Table 3:** Comparison between the designed antenna and other antennas reported on literature. (#Sw: Number of switches, #Pt: Number of ports, BW: Bandwidth (GHz), Av. G: Average Gain (dB), # Beams: Number of beams, Steer: Beam steering angle (deg), Size: Occupied volume (wavelengths at central frequency), EVM: Mean EVM (%) at a given input power.

Ref.	#Sw	#Pt	BW	Av G	#Beams	Steer	Size	EVM
This work	12	2	3.3–3.8	6.5	9	40	0.76×0.87×0.10	4 at 35 dBm
[30]	17	1	3.1–3.9	9	3	30	1.05×1.05×0.25	6 at 30 dBm
[29]	49	1	2.4–2.6	7.5			1.38×0.71×0.007	16 at 5 dBm
[28]	12	1	2.4–2.5	6.5	9	30	0.80×0.74×0.10	NA
[40]	60	1	2.4–3.0	4	9	30	2.16×1.08×0.05	NA

## Data Availability

The datasets generated during the current study are available from the corresponding authors on reasonable request.

## Abbreviations

The following abbreviations are used in this manuscript:

mmW	Millimeter Waves
RA	Reconfigurable Antenna
PL	Parasitic Layer
DUT	Device Under Test
TOI	Third Order Intercept
IP3	Third Harmonic Intercept Point
1dB CP	1 dB Compression Point
IMD	Inter-Modulation Distortion
EVM	Error-Vector Magnitude
IMPM	Internal Multi-Ports Method
LSOPS	Large Signal Operating Point Stimuli
RFS	RF Stimulus
DCS	DC Stimulus
